# iPhone 4s Photoplethysmography: Which Light Color Yields the Most Accurate Heart Rate and Normalized Pulse Volume Using the iPhysioMeter Application in the Presence of Motion Artifact?

**DOI:** 10.1371/journal.pone.0091205

**Published:** 2014-03-11

**Authors:** Kenta Matsumura, Peter Rolfe, Jihyoung Lee, Takehiro Yamakoshi

**Affiliations:** 1 College of Science and Engineering, Kanazawa University, Kanazawa, Ishikawa, Japan; 2 Department of Automatic Measurement and Control, Harbin Institute of Technology, Harbin, Heilongjiang, China; 3 Oxford BioHorizons Ltd., Maidstone, Kent, United Kingdom; 4 Graduate School of Natural Science and Technology, Kanazawa University, Kanazawa, Ishikawa, Japan; University of Adelaide, Australia

## Abstract

Recent progress in information and communication technologies has made it possible to measure heart rate (HR) and normalized pulse volume (NPV), which are important physiological indices, using only a smartphone. This has been achieved with reflection mode photoplethysmography (PPG), by using a smartphone’s embedded flash as a light source and the camera as a light sensor. Despite its widespread use, the method of PPG is susceptible to motion artifacts as physical displacements influence photon propagation phenomena and, thereby, the effective optical path length. Further, it is known that the wavelength of light used for PPG influences the photon penetration depth and we therefore hypothesized that influences of motion artifact could be wavelength-dependant. To test this hypothesis, we made measurements in 12 healthy volunteers of HR and NPV derived from reflection mode plethysmograms recorded simultaneously at three different spectral regions (red, green and blue) at the same physical location with a smartphone. We then assessed the accuracy of the HR and NPV measurements under the influence of motion artifacts. The analyses revealed that the accuracy of HR was acceptably high with all three wavelengths (all *r*s > 0.996, fixed biases: −0.12 to 0.10 beats per minute, proportional biases: *r* = −0.29 to 0.03), but that of NPV was the best with green light (*r* = 0.791, fixed biases: −0.01 arbitrary units, proportional bias: *r* = 0.11). Moreover, the signal-to-noise ratio obtained with green and blue light PPG was higher than that of red light PPG. These findings suggest that green is the most suitable color for measuring HR and NPV from the reflection mode photoplethysmogram under motion artifact conditions. We conclude that the use of green light PPG could be of particular benefit in ambulatory monitoring where motion artifacts are a significant issue.

## Introduction

The measurement of heart rate (HR) and the recording of the peripheral pulse (PP) have long been established as fundamental components of both research and clinical investigation [1], [2]. More recently, PP detected optically has been further investigated and this has led to the establishment of normalized pulse volume (NPV) as a valuable index for a variety of applications [3], [4]. HR and NPV, singly or in combination, have been used in, for example, the evaluation of stress and emotion [5], [6], [7], the assessment of cardiovascular health [8], [9], and for studying exercise intensity [10], [11]. Traditionally the measurement of these two variables has required conventional dedicated instruments, such as an electrocardiograph (ECG) and instruments for photoplethysmography (PPG). However, the recent emergence of the smartphone, which combines advanced computing power with its communication features, has offered an interesting and convenient alternative. The potential for physiological monitoring with a smartphone has been demonstrated by measuring the reflection mode finger-PPG, or pulse wave, with its embedded pseudo-white color flash light emitting diode (LED) as a light source and the complementary metal oxide semiconductor (CMOS) camera as a photo sensor [12], [13], [14]. HR (to be precise, pulse rate) and NPV can then be calculated from the PPG waveform. The measurement accuracy against dedicated devices at rest and during the conduct of prescribed tasks under well-controlled laboratory conditions has been reported [15], [16]. However, the performance of the smartphone approach under the influence of motion artifact is yet to be examined [16].

It is well recognized that motion artifact is a potential source of error with all PPG measurement systems [17], and this is also the case when using a smartphone for this purpose. In contrast to the application of PPG under strict laboratory conditions, when used in ambulatory subjects, in whom the smartphone approach might be expected to make a significant contribution [18], motion artifact seems inevitable. An important factor in the creation of movement artifact is that of the need to establish reliable and stable contact between the subject’s finger and the PPG light source and light detector. With conventional PPG systems the light source-detector sensors can be attached firmly and stably to the finger [15], whereas, the use of the smartphone requires the subject, or user, to grip the device in their hand and press and hold the tip of their index finger in contact with the smartphone camera, which is more problematic. Thus, for reliable use it is essential for smartphone PPG measurement to incorporate countermeasures against motion artifact.

Of direct relevance to this is the choice of light wavelength or color when deriving the photoplethysmogram with a smartphone. The CMOS camera simultaneously detects blue, green, and red light at essentially the same physical location, and all of these colors have been used for recording the PPG signals [13]. The simplicity of this approach for achieving multi-wavelength PPG is in contrast to the complexity of dedicated instruments [19], [20]. It has been reported that the PPG resistance to motion artifact can vary according to the wavelength used. For example, HR derived from laboratory green light PPG, using a 525 nm wavelength LED as a light source and a photo-diode (PD) as a photo sensor, has shown higher resistance to motion artifact than that from standard near-infrared light PPG [21]. This superiority of laboratory green light PPG among PPG with three primary light colors was also observed in our preliminary study [22]. A possible explanation for this finding is that near-infrared light has deeper penetration into the tissue, thereby probing those regions that are more subject to motion artifact [23], [24], [25].

Despite the growth in the interest of smartphone-based PPG for physiological applications, to date there has been no reported study to investigate which color (red, green, or blue) is the most suitable for HR and NPV measurement under motion artifact conditions. Conclusions about this important issue can not be easily extrapolated from earlier experience with conventional laboratory PPG instruments, since the two approaches are quite different in many respects. These differences particularly relate to the configuration and type of light source and detector and the precise manner in which they are interfaced to the finger. The fact that the optical configuration of the smartphone camera allows simultaneous recording of multiple PPGs at essentially the same anatomical location is novel. Therefore, in the present study, we measured red, green, and blue light PPGs using a smartphone while adding motion artifacts, and then compared the agreement of HR and NPV measurements derived from these reflection mode PPGs with those from motion artifact free conventional laboratory devices. We also compared signal-to-noise (S/N) ratio of all three color PPGs during baseline and while adding motion artifact. Our purpose was to clarify which light color is the most suitable for measuring HR and NPV when using a smartphone to perform PPG under the influence of motion artifact.

## Methods

### Ethics Statement

This study was approved by the ethical committee of the Faculty of Medicine of Kanazawa University (May 18, 2011, No.9) and conducted according to the principles expressed in the Declaration of Helsinki. Written informed consent was obtained from every participant after we had provided them with a complete description of the study.

### Participants

A total of 12 undergraduate male volunteer students, recruited through flyers, aged 20.6 ± 0.76 (*SD*) years, participated in this study. Because no effect size is available from previous studies, the sample size *n* = 12 was determined arbitrarily so that it should be multiples of the number of counterbalancing orders ( =  6) and equal to our previous smartphone study in order to compare the results directly. Inclusion criteria were having no history of or current cardiovascular disease, and not taking any prescription medication. Participants were asked in advance to refrain from any medication from the previous day of laboratory testing and, for 2 hours before laboratory testing, to avoid consumption of food and caffeine-containing substance, intense physical activity, and smoking.

### Procedure

**Figure 1:**
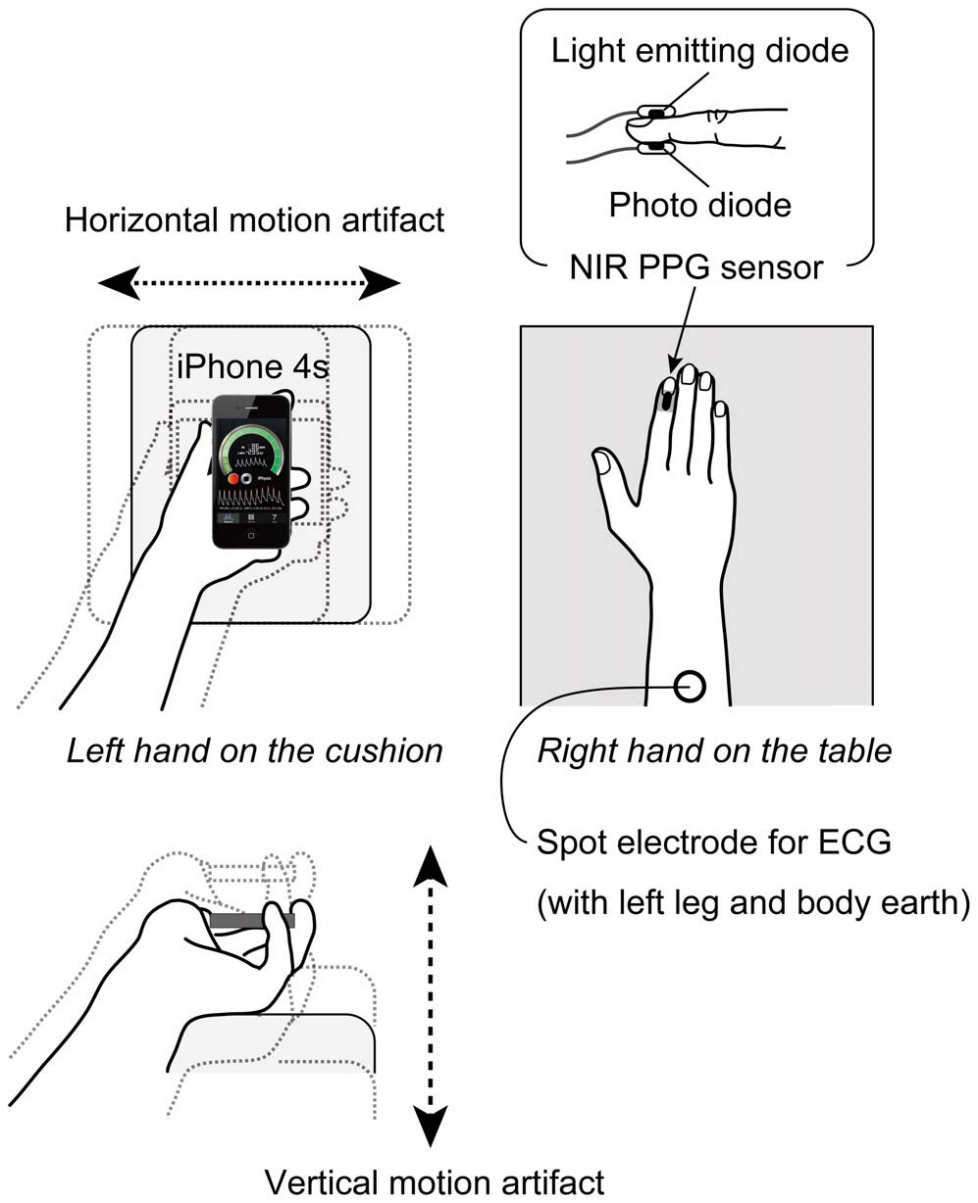
Schematic drawing of the experimental setup. The directions of the horizontal and vertical added motions are also shown. NIR  =  near-infrared, PPG photoplethysmogram, and ECG  =  electrocardiogram.

The experiment was conducted in a sound-attenuated experimental room, maintained at a temperature of 24−26 °C. The participants sat in a chair with their right hand on a desk, and the sensors and instruments required for conventional laboratory measurements were attached to the body and right index finger (Figure 1). Then, the subjects were asked to hold the iPhone 4s (Apple, Inc.) in their left hand, keeping their left index finger on the CMOS camera and the front screen vertical on a firm cushion on their knee. The subjects were asked to grip the iPhone sufficiently firmly to ensure that the finger-iPhone contact was essentially constant despite the existence of motion artifact. The height of the chair was adjusted so that both hands were at heart level.

**Figure 2:**
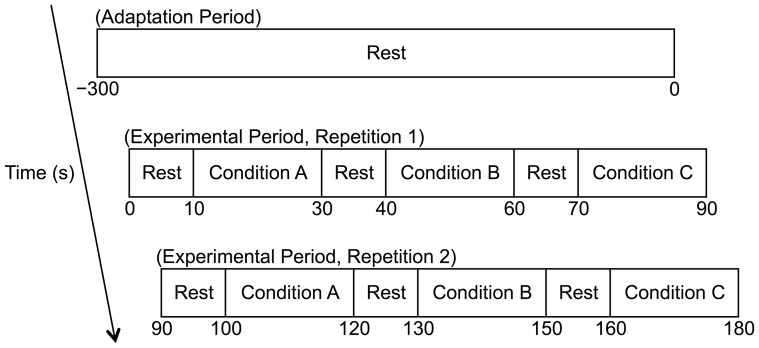
Experimental protocol. The combination of the condition (A, B, C) is assigned to one of the possible orders of the three motion artifact conditions. That is, (BL, HMA, VMA), (BL, VMA, HMA), (HMA, BL, VMA), (HMA, VMA, BL), (VMA, BL, HMA), or (VMA, HMA, BL). The orders were counter-balanced across the participants. BL  =  baseline, HMA  =  horizontal motion artifact, and VMA  =  vertical motion artifact.

After a careful check of the adequacy of finger-iPhone contact by adding a similar motion artifact used in the experiment as described below, a 5-min adaptation period was followed by a 3-min experimental period (Figure 2). In the adaptation period, the participant sat quietly with the experimenter standing in front of them and diagonally to the left. In the experimental period, the participant also sat quietly, but the experimenter shook by hand the cushion, on which the left hand with the iPhone 4s was placed, with a rhythmical motion at about 6 Hz to add motion intentionally only to the participant’s left hand and arm (Figure 1). The shaking intensity was determined so that visible noise was seen on the recorded PPG waveform. The frequency of 6 Hz was chosen because the main bandwidth of the PPG power spectrum is usually less than 5 Hz [26], [27], including the HR signal around 1 Hz, so it is easy to separate motion artifact from other sources in the PPG power spectrum. In this period, there were two motion artifact conditions; horizontal motion artifact (HMA) and vertical motion artifact (VMA), and one baseline (BL) condition. In the HMA and the VMA, the experimenter shook the cushion horizontally and vertically, respectively (Figure 1). In the BL, the experimenter simply held the cushion motionless. The order of HMA, VMA, and BL, were counter-balanced across participants (Figure 2). A set of these three conditions was repeated once again in the same order. Each 20-s condition was separated by 10-s rest periods.

### Apparatus and Measures

#### Laboratory devices

The reference, motion artifact free, beat-by-beat HR was calculated using the R-R interval of the Lead II electrocardiogram (ECG). This ECG was obtained using disposal electrodes connected to standard bioamplifiers [28] built in Kanazawa University. R peaks of the ECG were detected off-line using a local maximum search algorithm with the parameters of threshold peak value and non-detecting time along the time axis.

The reference, motion artifact free, beat-by-beat NPV was calculated by dividing the foot-to-peak amplitude of the alternating-current (AC), or pulsatile, component of the finger PPG by the direct-current (DC) component [3], [4]. NPV is an index of α-adrenalin mediated sympathetic activity, in other words, vascular tone, and is a measure that is in proportion to pulsatile arterial volume. The foot-to-peak amplitudes of the AC components, necessary to calculate NPV, were detected off-line using a paired local minimum and maximum search algorithm, with the parameters of minimum minimum-to-maximum amplitude, maximum minimum-to-maximum interval, and non-detecting time along the time axis. The reference PPG was measured in the transmission mode using an 810 nm near-infrared LED and a photodiode, placed on opposite sides of the tip of the right index finger. The sensors and an amplifier to which the sensors were connected were built in Kanazawa University [3]. A logarithmic transformation was applied to the NPV values to normalize the distribution.

All signals were sampled using an A/D converter at a rate of 1 kHz with a resolution of 16 bits, and stored digitally in a computer.

#### Smartphone

Red, green, and blue PPGs were measured simultaneously from the left index finger using an iPhone 4s (Apple, Inc.) within which the iPhysioMeter application [15] was installed. iPhysioMeter is a program that enables iOS (Apple, Inc.) devices with an LED flash light (i.e., so far, iPhone 4s and 5, and iPod touch) to measure and store the PPGs derived from all of the three colors and the three-axis g-forces simultaneously at a sampling frequency of 30 Hz (30 fps). No interpolation technique was employed. This application is available at iTunes App Store (Apple, Inc.) and is free.

The PPG measurement with iPhysioMeter uses the reflection mode, in which the pseudo-white color flash LED as a light source and the CMOS camera as a photo sensor are positioned essentially side-by-side. At the beginning of the measuring sequence, the CMOS camera is set to video capture mode with 192×144 pixels resolution, and each pixel has 8 bits depth for the red, green, and blue signals. Furthermore, the white balance, exposure, and focus mode of the CMOS camera were set via the iPhysioMeter ‘Settings’ control to “Locked”, “ContinuousAutoExposure”, and “Locked”, respectively [29]. These settings were chosen to keep the relative sensitivity to each light color constant whereas the total sensitivity of the CMOS camera to all light colors is then adjusted automatically by the iOS (CMOS camera embedded with iOS devices has a mechanical adjustment system for exposure). Once measurement has begun, each video frame captured by the CMOS camera is averaged among all of the pixels by each light color to produce a red, green, and blue light intensity value at the rate of 30 frames per second (fps).

Although iPhysioMeter is equipped with an on-line beat-by-beat HR and NPV auto analysis function *ab initio*, it is only designed to analyze the PPG from one of the three colors as set beforehand. So, beat-by-beat HRs and NPVs derived from the PPG from each color were calculated off-line using a Mac computer port of iPhysioMeter’s beat-by-beat HR and NPV auto analysis program. In these applications, foot and peak values of each light color AC component were detected using a paired local minimum and maximum search algorithm with the parameters of minimum minimum-to-maximum amplitude, maximum minimum-to-maximum interval, and non-detecting time along the time axis, whose parameters were adaptively changed with reference to the trend of the preceding 10-s period. The NPV values for each light color were calculated by dividing the foot-to-peak amplitudes of the AC component of the corresponding PPG signal by their direct-current (DC) components, respectively [3]. The HR values for each light color were calculated using the peak-to-peak intervals, these peaks being the same as those used in the NPV calculation, of corresponding PPG signals, respectively. Values departing significantly from the preceding 10-s period, defined as those contributing to increase the SD of the period above 8.0 beats per minute (bpm) for HR or 0.25 arbitrary units (a.u.) for lnNPV, respectively, are judged as outliers.

The S/N ratio was calculated from the raw red, green, and blue PPG waveforms using the following formula: S/N ratio  =  log_10_ (Signal Power / Noise Power). Here, signal and noise power are derived as the integrated power over a ± 0.5 Hz bandwidth from mean HR (Hz) that was calculated from R-R interval and peak motion artifact (Hz) around 6 Hz within the PPG power spectrum, respectively. The Power spectra of the PPGs were calculated using the fast-Fourier-transform with BIMUTAS II (Kissei Comtec, Inc.). A bandwidth of 1 Hz was used to include the whole of the corresponding power to take account of the fluctuations of the center frequencies of HR and motion artifact, in line with earlier published work [30].

Three-axis accelerations (x- and y-: horizontal, z-: vertical) were measured by the acceleration sensor contained within the iPhone 4s. The iPhysioMeter application reads the sensor output at the same sampling frequency as that used for the PPGs, that is, 30 Hz.

### Data Reduction

The HR values derived from the ECG and the red, green, and blue light PPG waveforms and the lnNPV values from near-infrared light PPG and red, green, and blue light PPG were averaged for each 20-s period, and further averaged across two repetitions to obtain the values for BL, HMA, and VMA. Those values judged as outliers were removed before averaging.

The S/N ratios calculated from the red, green, and blue light PPG signals for each 20-s period were averaged across repetitions to obtain the BL, HMA, and VMA values.

The amplitudes of the three-axis acceleration signals were averaged for each 20-s period, and further averaged across repetitions to obtain the BL, HMA, and VMA values. Then, x- and y-axis amplitudes were synthesized using the Pythagoras theorem to obtain the horizontal amplitudes of the BL, HMA, and VMA values.

### Statistical Analysis

HR and lnNPV values for each measurement (reference, red, green, and blue light PPG) and condition (BL, HMA, and VMA) were analyzed using a series of separate two-way repeated-measures analysis of variance (ANOVA), after having checked the normality of the distribution by Kolmogorov-Smirnov tests. The Greenhouse-Geisser correction was applied to the degree of freedom where appropriate. For post-hoc comparison, Tukey HSD tests were used.

To evaluate the agreement of HR and lnNPV measurements derived from each of the three PPGs with the corresponding reference values, geometric mean regression [31] and Bland-Altman plot [32] analyses were used. In the former, an intercept (fixed bias), slope (proportional bias), and correlation coefficient (Pearson’s r) were calculated, whereas in the latter, the mean of the differences (fixed bias) and correlation coefficient between averages and differences (proportional bias) were calculated. In these analyses, a total of 72 data points (three conditions × two repetitions × 12 participants) were used.

S/N ratios for each color (red, green, and blue) and condition (BL, HMA, and VMA) were analyzed using a two-way repeated-measures ANOVA after having checked the normality of the distribution by Kolmogorov-Smirnov tests. The Greenhouse-Geisser correction was applied to the degree of freedom where appropriate. For post-hoc comparison, separate one-way repeated-measures ANOVAs were conducted when interaction was observed, and for its multiple comparison, Tukey HSD tests were used.

The differences between horizontal and vertical acceleration for each condition were compared using Wilcoxon signed-rank test.

Statistical analyses were performed using IBM SPSS Statistics 19 for Mac (IBM Inc.).

## Results

**Figure 3:**
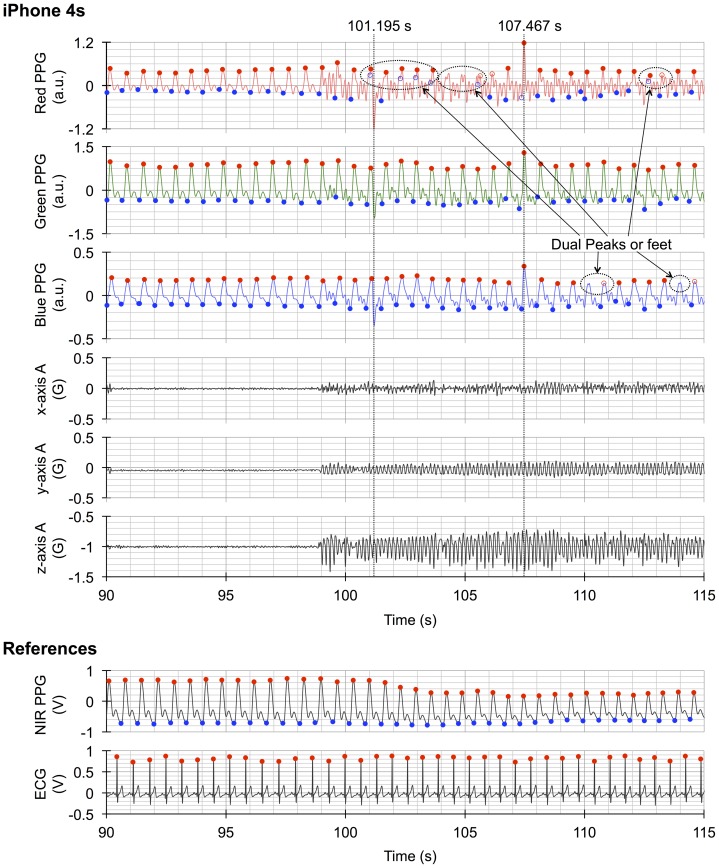
Recording of smartphone red, green and blue PPGs, 3-axis accelerations, and reference PPG and ECG. (**iPhone 4s**) The AC components of the red, green, and blue light smartphone PPGs, together with x-, y- (horizontal), and z- (vertical) axis acceleration (A); (**References**) The simultaneous recordings of reference, motion artifact free, near-infrared PPG and ECG. The left side and the right side of the chart correspond to in the absence and presence of motion artifact, respectively. Red and blue closed circles represent the peak and foot points identified by a beat-by-beat auto analysis algorithm incorporated in iPhysioMeter. Peak-to-peak intervals and foot-to-peak amplitudes are used to calculate heart rate (HR) and normalized pulse volume (NPV). Open circles identify outlier points, that may have arisen from misidentification and/or omission of an expected preceding point, are also judged by this algorithm. The differential effect of motion artifacts on the smartphone PPGs from the three light colors and the outputs of peak and foot detection are clearly shown. Dual peaks and feet in the PPG waveform due to motion artifact can exert a harmful influence on intact foot and peak detection. Here intense peaks in the three light color PPGs due to the change in iPhone - finger contact pressure, often accompanying motion artifact, are in sync at 101.195 and 107.467 s. PPG  =  photoplethysmogram, ECG  =  electrocardiogram.

**Figure 4:**
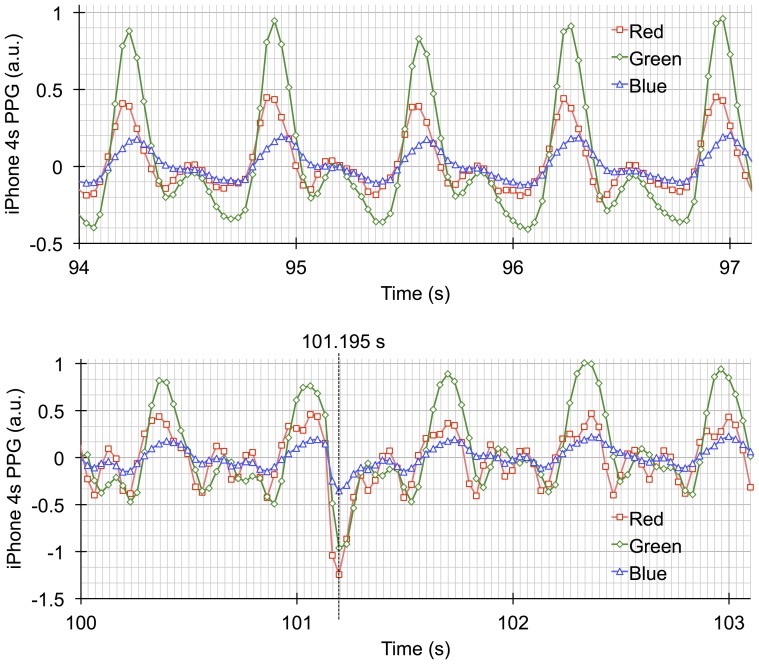
Expanded and overlaid recordings from Figure 3. The upper recording was made in the absence of artifact, whilst the lower recording was made during the addition of movement to the left hand. PPG  =  photoplethysmogram,

**Figure 5:**
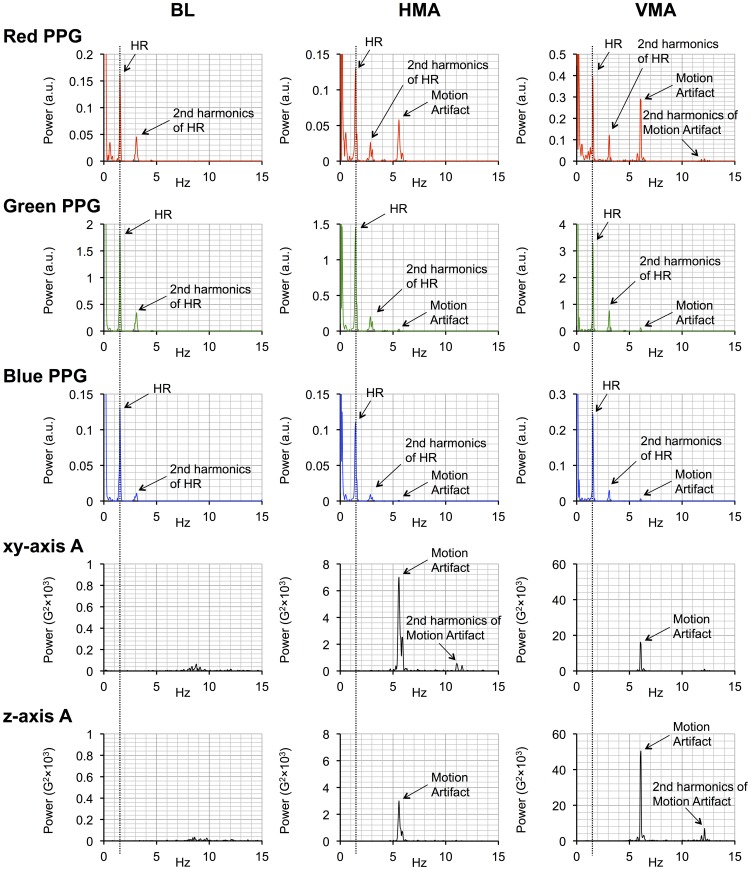
Typical example of smartphone PPG and acceleration power spectra by three conditions and three colors. The differential effect of motion artifacts on the PPGs from the three light colors is clearly shown. Three vertical dotted lines around 1.5-R intervals. BL  =  baseline, HMA  =  horizontal motion artifact, VMA  =  vertical motion artifact, PPG  =  photoplethysmography, HR  =  heart rare, and A  =  acceleration.

Figure 3 shows a typical example of the AC components of the three photoplethysmograms derived from the red, green, and blue light, together with x-, y- (horizontal), and z- (vertical) axis acceleration, and the simultaneous recording of the reference photoplethysmograms and the ECG (raw data are provided in Table S4). The chart clearly illustrates the presence of motion artifacts on the AC waveforms (right side) derived from the smartphone, which are not present in the absence of motion waveforms (left side) and in the reference photoplethysmograms and ECG recordings. More importantly, the differential effect of motion artifacts on the smartphone PPG signals for the three light colors are clearly shown. Figure 4 shows expanded and overlaid recordings from Figure 3. Figure 5 shows a typical example of smartphone PPG and acceleration power spectra for three motion artifact conditions and three light colors (individual data are provided in Table S5). These graphs clearly demonstrate the separation of motion artifact from other signal sources in the PPG power spectrum and the pure sine wave form of the motion-induced artifact.

### Motion artifact during BL, HMA, VMA

The median values and their ranges of horizontal and vertical acceleration during each condition, together with results of Wilcoxon signed-rank tests, are summarized in Table 1 (individual data are provided in Table S1). Because the Kolmogorov-Smirnov tests did not confirm the normality of the distribution in horizontal and vertical acceleration (not all *p*s > 0.05), we conducted nonparametric tests here.

**Table 1:** Median g-forces during three motion artifact conditions and results of statistical tests.

Condition	Acceleration (G)		Results of Wilcoxon Signed-rank Test	
	Horizontal	Vertical		
	Median	Median	*W*	*p*
	[Range]	[Range]		
BL	0.013	0.011	66	0.034
	[0.008−0.025]	[0.007−0.030]		
HMA	0.34	0.20	78	0.002
	[0.18−0.75]	[0.05−0.57]		
VMA	0.19	0.42	78	0.002
	[0.10−0.33]	[0.30−0.60]		

*Note.* BL  =  baseline, HMA  =  horizontal motion artifact, and VMA  =  vertical motion artifact.

### HR and lnNPV for reference and red, green, and blue light PPG during BL, HMA, and VMA

The mean values of HR and lnNPV for each measurement during each condition are summarized in Table 2 (individual data are provided in Table S2). Kolmogorov-Smirnov tests confirmed the normality of the distribution in all indices (all *p*s > 0.05). For HR, the repeated-measures ANOVA did not reveal any significant main effects of measurement, *F*(3, 33)  = 2.39, *p* = 0.119, *ε* = 0.63, *η*_p_^2^ = 0.18, and condition, *F*(2, 22)  = 0.25, *p* = 0.783, *η*_p_^2^ = 0.02, and measurement × condition interaction, *F*(6, 66) = 0.76, *p* = 0.502, *ε* = 0.40, *η*_p_^2^ = 0.06. For lnNPV, in contrast, the repeated-measures ANOVA revealed significant main effect of measurements, *F*(3, 33)  = 128.25, *p*<0.001, *η*_p_^2^ = 0.92, but not condition, *F*(2, 22)  = 1.64, *p* = 0.217, *η*_p_^2^ = 0.13, and measurement × condition interaction, *F*(6, 66)  = 2.73, *p* = 0.077, *ε* = 0.39, *η*_p_^2^ = 0.20. Subsequent post-hoc Tukey HSD test revealed that lnNPV derived from reference and green light PPG were higher than that from blue light PPG, and that lnNPV derived from blue light PPG was higher than that from red light PPG. This post-hoc result is also shown in Table 2.

**Table 2:** Mean heart rate (HR) and log-transformed normalized pulse volume (lnNPV) during three motion artifact conditions by different measurements.

Variables	Condition	Measurement			
		Reference	Red PPG	Green PPG	Blue PPG
		Mean (SD)	Mean (SD)	Mean (SD)	Mean (SD)
HR (bpm)	BL	69.9 (7.7)	69.8 (7.7)	70.0 (7.8)	69.9 (7.9)
	HMA	70.4 (8.2)	70.2 (8.3)	70.5 (8.3)	70.4 (8.6)
	VMA	70.3 (9.3)	70.3 (9.1)	70.4 (9.2)	70.5 (9.2)
lnNPV (a.u.)	BL	−4.24_a_ (0.69)	−5.98_c_ (0.62)	−4.26_a_ (0.61)	−5.22_b_ (0.44)
	HMA	−4.26_a_ (0.71)	−5.93_c_ (0.65)	−4.31_a_ (0.66)	−5.24_b_ (0.52)
	VMA	−4.29_a_ (0.67)	−5.88_c_ (0.66)	−4.24_a_ (0.65)	−5.12_b_ (0.59)

*Note.* BL  =  baseline, HMA  =  horizontal motion artifact, VMA  =  vertical motion artifact, PPG  =  photoplethysmography, bpm  =  beats per minute, and a.u.  =  arbitrary units.

Means sharing a common subscript are not statistically different at *α*  =  0.01 according to the two-way repeated-measures analysis of variance (ANOVA) and Tukey HSD procedure.

Although the BL period was very short, we also calculated the mean and SD of the standard deviation of normal-to-normal RR intervals (SDNN), which is one of the basic time domain measures of heart rate variability. The SDNN of the reference ECG and the red, green, and blue light PPGs during BL were 36.3 (15.5), 41.9 (14.2), 41.2 (14.0), and 44.8 (16.0) ms, respectively (individual data are provided in Table S7).

### Agreement of HR and lnNPV between PPGs from red, green, and blue light and from the reference

**Figure 6:**
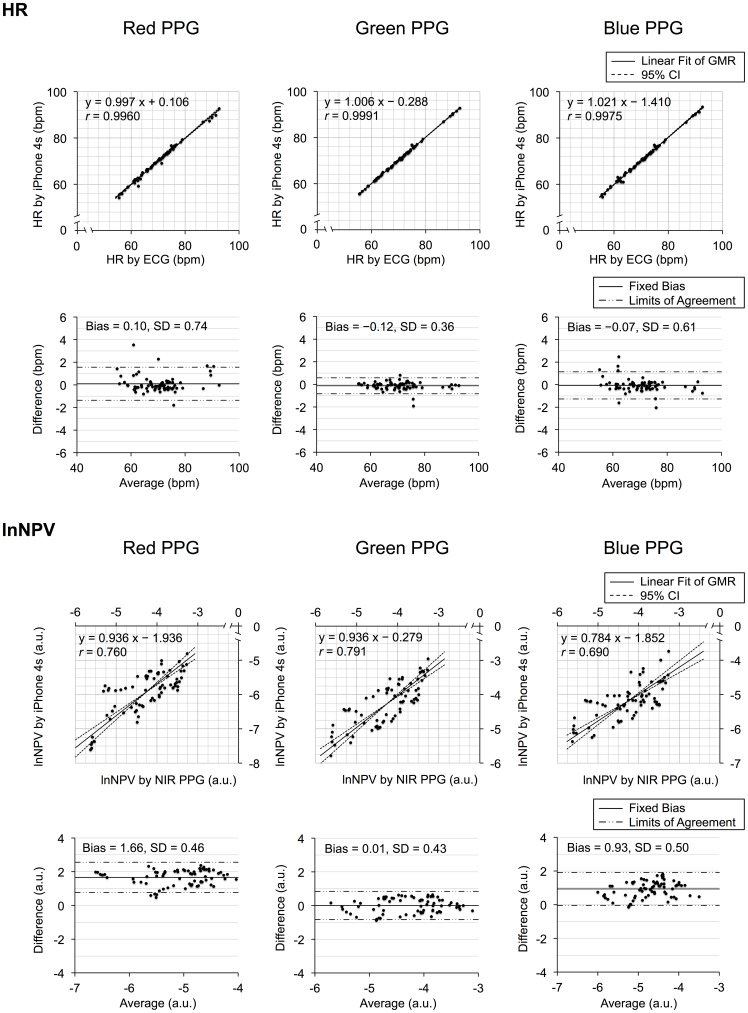
The agreement of HR and lnNPV measured by the smartphone PPG and corresponding references. Each section of heart rate (**HR**: Top) and log-transformed normalized pulse volume (**lnNPV**: Bottom) includes the three pairs of scatterplot (Upper) and Bland-Altman plot (Lower) measured by red (Left), green (Center), and blue (Right) light smartphone PPG against those by the corresponding reference devices among all of the 72 data pairs (three conditions × two repetitions × 12 participants). ECG  =  electrocardiography, PPG  =  photoplethysmography, NIR  =  near-infrared, GMR  =  geometric mean regression, bpm  =  beats per minute, and a.u.  =  arbitrary units.

Scatter plots of paired HRs and lnNPVs derived from red, green, and blue PPG against the corresponding reference, together with their Bland-Altman plots, are shown together in Figure 6 (individual data are provided in Table S3). The results of geometric mean regression and Bland-Altman analysis are summarized in Table 3.

**Table 3:** Outcomes of geometric mean regression analyses and Bland-Altman plots.

Variables	PPG Color	Geometric Mean Regression			Bland-Altman Plot		
		Slope	Intercept	*r*	Mean	SD	*r*
		[95% CI]	[95% CI]	[95% CI]	[95% LOA]		[95% CI]
HR (bpm)	Red	0.997	0.106	0.9960	0.10	0.74	0.03
		[0.976, 1.019]	[−1.407, 1.587]	[.9935,.9975]	[−1.36, 1.56]		[−0.20, 0.26]
	Green	1.006	−0.288	0.9991	−0.12	0.36	−0.14
		[0.996, 1.016]	[−1.012, 0.429]	[.9985,.9994]	[−0.83, 0.58]		[−0.36, 0.10]
	Blue	1.021	−1.410	0.9975	−0.07	0.61	−0.29
		[1.004, 1.038]	[−2.619, −0.221]	[.9961,.9985]	[−1.27, 1.13]		[−0.48, −0.06]
lnNPV (a.u.)	Red	0.936	−1.936	0.760	1.66	0.46	0.10
		[0.803, 1.093]	[−2.507, −1.270]	[.641,.843]	[0.77, 2.56]		[−0.13, 0.32]
	Green	0.936	−0.279	0.791	0.01	0.43	0.11
		[0.809, 1.082]	[−0.819, 0.345]	[.685,.864]	[−0.83, 0.84]		[−0.13, 0.33]
	Blue	0.784	−1.852	0.690	0.93	0.50	0.32
		[0.660, 0.931]	[−2.379, −1.225]	[.545,.795]	[−0.04, 1.90]		[0.10, 0.51]

*Note.* HR  =  heart rate, lnNPV  =  log-transformed normalized pulse volume, PPG  =  photoplethysmography, CI  =  confidential interval, LOA  =  limits of agreement, bpm  =  beats per minute, and a.u.  =  arbitrary units.

Each *n*  =  72 (three conditions × two repetitions × 12 participants).

### S/N ratio for red, green, and blue light PPGs during BL, HMA, and VMA

**Figure 7:**
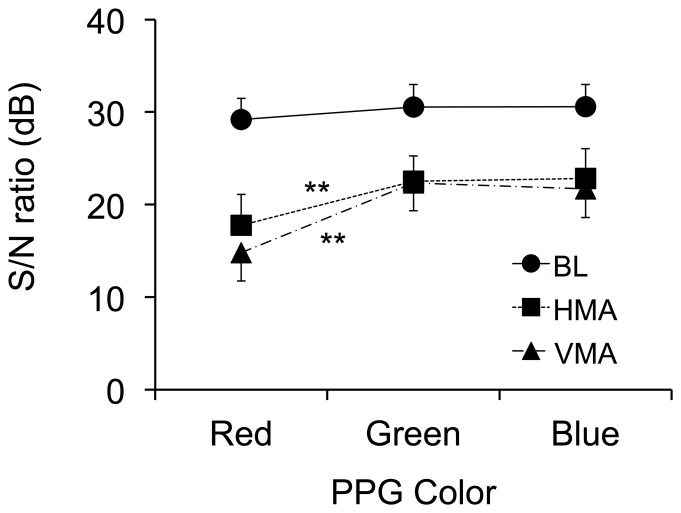
Mean values of S/N ratio by three motion artifact conditions and three colors. S/N  =  signal-to-noise, BL  =  baseline, HMA  =  horizontal motion artifact, VMA  =  vertical motion artifact, and PPG  =  photoplethysmography. Vertical bar represent for SEM. ^**^ *p*<0.01.

The mean values of S/N ratios for each PPG color during each condition are shown in Figure 7 (individual data are provided in Table S6). The Kolmogorov-Smirnov tests confirmed the normality of the distribution in all indices (all *p*s > 0.05). The repeated-measures ANOVA revealed significant main effect of color, *F*(2, 22)  = 11.20, *p*<0.001, *η*_p_^2^ = 0.50, and condition, *F*(2, 22)  = 34.56, *p*<0.001, *η*_p_^2^ = 0.76, and color × condition interaction, *F*(4, 44)  = 11.43, *p*<0.001, *η*_p_^2^ = 0.51. Subsequent post-hoc tests revealed significant simple main effect of color during HMA, *F*(2, 22)  = 7.81, *p* = 0.003, *η*_p_^2^ = 0.42, VMA, *F*(2, 22)  = 25.54, *p*<0.001, *η*_p_^2^ = 0.70, but not BL, *F*(2, 22)  = 1.02, *p* = 0.378, *η*_p_^2^ = 0.08. In addition, subsequent post-hoc Tukey HSD test revealed that S/N ratios derived from green and blue light PPGs were higher than that from red PPG in both HMA and VMA condition.

## Discussion

Our goal was to clarify, when using a smartphone to perform PPG under the influence of motion artifact, which light color - red, green, or blue - is the most suitable for measuring HR and lnNPV. To address this, during baseline and while adding motion of the smart phone to create artifact in the PPG signal, we examined the agreement of HR and NPV measurements derived from the photoplethysmograms recorded with these three colors and from the reference methods, and the signal-to-noise (S/N) ratio of the PPGs from the three light colors. As a result, firstly, the levels of agreement between paired HR values were quite high and comparable for the data from all three light colors. All paired data exhibited nearly 1.00 correlation coefficients and negligible fixed and proportional biases, though the best agreement was observed for the green light PPG. Secondly, the agreement for the lnNPV measurements was the best with the green light PPG. The results from green light PPG showed negligible fixed and proportional biases, whereas the results from red and blue light PPG exhibited fixed and both fixed and proportional biases, respectively. Thirdly, the S/N ratio was higher with green and blue light PPG than with the red light PPG during both HMA and VMA, though comparable during BL. Taken together, these findings suggest that green is the most suitable light color among the three possible colors available from the smartphone for both HR and lnNPV measurement under motion artifact.

HR values calculated from the PPG signals from all three light colors were accurate in that all of the relevant paired data exhibited nearly 1.00 correlation coefficients. However, as already mentioned above, the three differed in their absolute accuracy. Several factors could be responsible for creating these differences. Firstly, the waveforms of the photoplethysmograms from the three light colors exhibit some differences. In the left side of Figure 3, showing recordings made in the absence of motion artifact, there is a suggestion of temporal differences between the identified peaks; this can be better appreciated in Figure 4, where the time-scale has been expanded. For example, the first peaks seen in the red, green, and blue PPG signals in the absence of motion artifact in Figure 4 occur at 94.190 s, 94.221 s (1 fps after 94.190 s), and 94.256 s (2 fps after 94.190 s), respectively. As already mentioned in the introduction, blue light PPG probes the shallowest arterioles, which are the most distal of the three vascular regions to be probed. It is known that the more distal the PPG probing region is the more the waveform loses its edge [e.g., 26]. This is also clearly seen in Figure 5, where the ratio of the 1st to 2nd harmonics of the HR power decreases in accordance with the decreases in the tissue penetration depth, which is known to be dependant on wavelength.

The second matter of relevance is that, despite the temporal and penetration depth phenomena, there can be occasions when the effect of motion artifact on each of the three photoplethysmograms can be essentially synchronized. This means that, in the above example, if motion artifact produces an intense peak at 94.155 s on the PPG signal (this point is 1, 2, and 3 fps before red’s peak at 94.190 s, green’s at 94.221 s, and blue’s at 94.256 s, respectively), each peak of the three light colors would be detected not at each actual peak (red’s 94.190 s, green’s 94.221 s, and blue’s 94.256 s peak, respectively), but at the time of this intense peak (at 94.155 s). That is, the peak shift intervals of red, green and blue are 1, 2, and 3 fps, respectively, and thus not constant among the three colors. In this way, HRs derived from each light color photoplethysmogram are differentially influenced by a motion artifact near the actual peak points. Such phenomena actually occurred at 101.195 s in Figures 3 and 4, and the peaks of the three light color photoplethysmograms are synchronized here.

The third and final issue is that, whether such peak shifts really occur or not depends on the tolerance of each light color PPG to the motion artifacts. In this regard, it seems reasonable to conclude that the HR accuracy derived from red light PPG is inferior to that from green light PPG. This is because the S/N ratio of PPG is lower in red light than in green light during both HMA and VMA, thus the shift of peak would occur more in red light PPG than in green light PPG. On the other hand, this view can not offer a reasonable explanation for the difference we have found between green and blue light PPG data, because the S/N ratio of PPG using these two colors were comparable. However, the apparently blunted leading edge seen with blue light PPG possibly causes the worsening of the tolerance to motion artifact, especially around the peaks. In fact, such a tendency is clearly observed in Figure 3 as a dual peak only in blue light color PPG around at 110, and 114, s. Although many of such dual peaks are judged as outliers by the auto analysis algorithm within iPhysioMeter, as shown in Figure 3, it is difficult to eliminate all such points, and thus there is room for some errors to be produced. Since this is the case, the HRs calculated from each light color are differentially influenced by the motion artifacts, and thus their accuracies are different from one another.

The S/N ratio was higher in the blue and green light PPGs than in the red light PPGs during both HMA and VMA. This tendency is consistent with our preliminary study conducted using laboratory devices where 470 (blue), 530 (green), and 645 (red) nm wavelength LED as a light sources and the photo-diode (PD) as a photo sensor [22]. Considering that the S/N ratios obtained with all three colors were comparable during BL and were derived simultaneously at the same anatomical location, such a pattern most likely arises from the relationship between their probing depth and the anatomical origin of the motion artifact [25]. According to Maeda et al. [25], Giltvedt et al. [24], Lindberg and Oberg [33], and Ugnell and Oberg [34], green PPG most likely probes ascending arterioles and/or superficial plexus arterioles located in the upper region of the reticular dermis. It is known that light of longer wavelengths has deeper penetration into the biological tissue than light of shorter wavelengths, and that the penetration depth of green light approximates more closely to that of blue than that of red [23], [24]. Therefore blue light PPG is likely to probe superficial plexus arterioles in the upper part of the reticular dermis, whereas red light PPG most likely probes the deep plexus arterioles located in the lower part of the reticular dermis or small arteries *sub cutis*. However, despite these differences in the extent to which arterioles are probed, both blue and green light PPGs probe arterioles embedded in the same anatomical structure, the upper part of the reticular dermis, and as such are likely show a similar response to the motion. This interpretation seems to provide a probable explanation why blue and green light PPGs show the comparable resistance to motion artifact. On the other hand, deep arterioles or arteries probed by red light are measured via dermis that has a lower Young modulus, and as such is unsurprisingly easy to displace or compress. Thus, it seems reasonable that red light PPG shows lower resistance to motion artifact compared to blue and green light PPG. This suggests that the findings from dedicated devices can possibly be extended to smartphone PPG measurement regardless of their significant differences in configuration and type of light source and detector, and the precise manner in which they are interfaced to the finger.

A further word of caution is necessary with regard to the general description of the light used in PPG as being “red”, “blue”, or “green”. Where dedicated light sources and detectors have been used, including lasers or LEDs, the wavelength has been specified with some precision. For example, in studying depth discrimination in laser Doppler skin blood flow measurement [35], green and red light from helium-neon lasers had wavelengths of 543 nm and 632.8 nm respectively, whilst near-infrared light from a laser diode was reported to have a wavelength of 780 nm. In the case of the smartphone, however, a CMOS image sensor is used and such devices have comparatively broad spectral characteristics. For example typical devices may have full width half maximum values of 150 nm or more, and there can therefore be significant overlap in the red, green and blue bands. This significantly complicates the interpretation of possible wavelength-dependant penetration depth effects, as well as the comparison between results of published studies.

Fixed bias in green light PPG was *ipso facto* zero and correlation coefficients were nearly 0.8 in this study. These results are somewhat different from the previous study conducted by Matsumura and Yamakoshi [15], which showed a relatively large fixed bias in lnNPV (0.87 a.u.; i.e. lnNPV from smartphone green light PPG was smaller than that from reference near-infrared light PPG), though within the limits of agreement, and relatively low correlation coefficients (*r* = 0.432) compared to the present study. These differences are plausibly due to the manner of holding the smartphone. That is, in the study of Matsumura and Yamakoshi [15], the participants held the smartphone relatively loosely whereas in the present study they held it more firmly so that the finger-smartphone contact was constant. This modification was made simply in consideration of the nature of the present experiment; i.e. adding motion artifact. However, as a consequence of this, it is possible that finger-iPhone contact pressure was increased, which in turn could cause a local decrease in arteriolar pressure. Supporting this view, it is observed that the pulse wave in fact disappears when the finger is pressed very firmly against the smartphone image sensor. Overall, then, it is likely that NPV derived from green light PPG increased to the level of that from near-infrared light PPG because lnNPV is known to increase in accordance with local blood pressure decrease [36]. Thus, to achieve successful performance of iPhysioMeter the finger-iPhone contact should be maintained relatively constant during operation, with firmness to the extent that it does not completely occlude the artery. The net effect should be that correlation coefficients are increased via the decrease in the fluctuation in finger-iPhone contact pressure that is considered to serve as a random error in measurement [15].

Our results revealed the superiority of green light PPG over both red and blue light PPG. However, it has been reported that additional value can be obtained from red and blue light PPG using the smartphone in that estimates of blood SpO_2_ can be derived from the red and blue lnNPV data [14]. Our results also showed that the S/N ratios of the three light color PPGs were comparable during BL; that is, all were approximately 30 dB, so the measurement under such resting BL condition would be acceptable with any of the three light colors. However, in the case of measurements being made during normal daily-life, acceleration should also be measured whenever possible in that unpredictable motion can occur. In fact, recent smartphones are usually equipped with an acceleration sensor and with the computational power to calculate S/N ratio.

### Limitations of the study, open questions, and future work

There are some limitations in this study. Firstly, the population of the participants was limited in terms of age range, skin tone, and sample size was relatively small. Age could be important since it is well known that the skin becomes stiffer and less flexible with increasing age [37]. Nevertheless, in some previously published PPG motion artifact studies, the populations of participants were exclusively limited to young men [21], [22], [25]. Furthermore, differences in skin tone as well as wavelength could also affect the tissue penetration depth; it can be shallower in pigmented individuals [23]. Thus, the generalizability of the results from the present study could be of interest. Secondly, the motion artifact used in this study was strictly controlled, and restricted to a rather limited spatial orientation and frequency. As a contrast to the approach used in the present study, experiments adding white noise having a flat power spectrum, could be of value. This is because in real ambulatory settings such as during exercise and running, there are many more kinds of motion artifact in terms of both orientation and frequency. Thirdly, because HR and NPV were the target measures in this study, each experimental period was set to 20 sec so as to give sufficient time to calculate these. However, such short recording periods makes standard heart rate variability calculations impossible, thus longer periods to allow the calculation of these measures would be preferable in future studies. Fourthly, we have used only one smartphone model, namely iPhone 4s, in the present study. So, replicating studies using other smartphones could be of value. Taken together, further studies dealing with these points are needed.

## Conclusions

Our results suggest that green light PPG is the most suitable color among the three colors tested here for smartphone HR and lnNPV measurement. This finding, in combination with known digital signal processing approaches to reduce motion artifact [17], [38] and/or robust feature detection algorithm [39], will help to achieve robust measurement in ambulatory settings, where motion-induced artifact is likely to be a significant issue.

## Supporting Information

Table S1 **Individual data of** **Table 1** **.**
(XLSX)

Table S2 **Individual data of** **Table 2** **.**
(XLSX)

Table S3 **Individual data of** **Table 3** **and** **Figure 6** **.**
(XLSX)

Table S4 **Raw data of** **Figure 3** **and** **4** **.**
(XLSX)

Table S5 **Individual data of** **Figure 5** **.**
(XLSX)

Table S6 **Individual data of** **Figure 7** **.**
(XLSX)

Table S7 **Individual data of SDNN during BL.**
(XLSX)

## References

[1] Parker KH (2009) A brief history of arterial wave mechanics. Med Biol Eng Comput 47: 111–118 10.1007/s11517-009-0440-5 19198914 PMC2644374

[2] Challoner AVJ (1979) Photoelectric plethysmography for estimating cutaneous blood flow. In: Rolfe P, editor. Noninvasive Physiological Measurements. London: Academic Press. pp. 125–151.

[3] Lee J, Matsumura K, Yamakoshi T, Rolfe P, Tanaka N, et al (2013) Validation of normalized pulse volume in the outer ear as a simple measure of sympathetic activity using warm and cold pressor tests: towards applications in ambulatory monitoring. Physiol Meas 34: 359–375 10.1088/0967-3334/34/3/359 23442846

[4] Sawada Y, Tanaka G, Yamakoshi K (2001) Normalized pulse volume (NPV) derived photo-plethysmographically as a more valid measure of the finger vascular tone. Int J Psychophysiol 41: 1–10 10.1016/S0167-8760(00)00162-8 11239692

[5] Hamer M, Tanaka G, Okamura H, Tsuda A, Steptoe A (2007) The effects of depressive symptoms on cardiovascular and catecholamine responses to the induction of depressive mood. Biol Psychol 74: 20–25 10.1016/j.biopsycho.2006.06.003 16860921

[6] McNally RJ, Lasko NB, Clancy SA, Macklin ML, Pitman RK, et al (2004) Psychophysiological responding during script-driven imagery in people reporting abduction by space aliens. Psychol Sci 15: 493–497 10.1111/j.0956-7976.2004.00707.x 15200635

[7] Matsumura K, Yamakoshi T, Noguchi H, Rolfe P, Matsuoka Y (2012) Fish consumption and cardiovascular response during mental stress. BMC Res Notes 5: 288 10.1186/1756-0500-5-288 PMC3414797 22695000

[8] Tanaka G, Yamakoshi K, Sawada Y, Matsumura K, Maeda K, et al (2011) A novel photoplethysmography technique to derive normalized arterial stiffness as a blood pressure independent measure in the finger vascular bed. Physiol Meas 32: 1869–1883 10.1088/0967-3334/32/11/003 22026968

[9] Kuvin JT, Patel AR, Sliney KA, Pandian NG, Sheffy J, et al (2003) Assessment of peripheral vascular endothelial function with finger arterial pulse wave amplitude. Am Heart J 146: 168–174 10.1016/S0002-8703(03)00094-2 12851627

[10] Matsumura K, Yamakoshi T, Yamakoshi Y, Rolfe P (2011) The effect of competition on heart rate during kart driving: A field study. BMC Res Notes 4: 342 10.1186/1756-0500-4-342 21906298 PMC3180469

[11] Yamakoshi T, Matsumura K, Yamakoshi Y, Hirose H, Rolfe P (2010) Physiological measurements and analyses in motor sports: a preliminary study in racing kart athletes. Eur J Sport Sci 10: 397–406 10.1080/17461391003699112

[12] Jonathan E, Leahy M (2010) Investigating a smartphone imaging unit for photoplethysmography. Physiol Meas 31: N79–83 10.1088/0967-3334/31/11/N01 20871134

[13] Jonathan E, Leahy MJ (2011) Cellular phone-based photoplethysmographic imaging. J Biophotonics 4: 293–296 10.1002/jbio.201000050 20815022

[14] Scully CG, Lee J, Meyer J, Gorbach AM, Granquist-Fraser D, et al (2012) Physiological parameter monitoring from optical recordings with a mobile phone. IEEE Trans Biomed Eng 59: 303–306 10.1109/TBME.2011.2163157 21803676 PMC3476722

[15] Matsumura K, Yamakoshi T (2013) iPhysioMeter: A new approach for measuring heart rate and normalized pulse volume using only a smartphone. Behav Res Methods 45: 1272–1278 10.3758/s13428-012-0312-z 23408381

[16] Gregoski MJ, Mueller M, Vertegel A, Shaporev A, Jackson BB, et al (2012) Development and validation of a smartphone heart rate acquisition application for health promotion and wellness telehealth applications. Int J Telemed Appl 2012: 696324 10.1155/2012/696324 22272197 PMC3261476

[17] Allen J (2007) Photoplethysmography and its application in clinical physiological measurement. Physiol Meas 28: R1–39 10.1088/0967-3334/28/3/R01 17322588

[18] Yamakoshi K (2013) In the Spotlight: BioInstrumentation. IEEE Rev Biomed Eng 6: 9–12 10.1109/RBME.2012.2227703 23193467

[19] Spigulis J, Gailite L, Lihachev A, Erts R (2007) Simultaneous recording of skin blood pulsations at different vascular depths by multiwavelength photoplethysmography. Appl Opt 46: 1754–1759 10.1364/AO.46.001754 17356618

[20] Gailite L, Spigulis J, Lihachev A (2008) Multilaser photoplethysmography technique. Lasers Med Sci 23: 189–193 10.1007/s10103-007-0471-9 17632746

[21] Maeda Y, Sekine M, Tamura T (2011) Relationship between measurement site and motion artifacts in wearable reflected photoplethysmography. J Med Syst 35: 969–976 10.1007/s10916-010-9505-0 20703691

[22] Lee J, Matsumura K, Yamakoshi K, Rolfe P, Tanaka S, et al. (2013) Comparison Between Red, Green and Blue Light Reflection Photoplethysmography for Heart Rate Monitoring During Motion. Conf Proc IEEE Eng Med Biol Soc: 1724–1727. doi:10.1109/EMBC.2013.6609852 24110039

[23] Anderson RR, Parrish JA (1981) The optics of human skin. J Invest Dermatol 77: 13–19 10.1111/1523-1747.ep12479191 7252245

[24] Giltvedt J, Sira A, Helme P (1984) Pulsed multifrequency photoplethysmograph. Med Biol Eng Comput 22: 212–215 10.1007/BF02442745 6738126

[25] Maeda Y, Sekine M, Tamura T (2011) The advantages of wearable green reflected photoplethysmography. J Med Syst 35: 829–834 10.1007/s10916-010-9506-z 20703690

[26] Kamal AA, Harness JB, Irving G, Mearns AJ (1989) Skin photoplethysmography—a review. Comput Methods Programs Biomed 28: 257–269. 2649304 10.1016/0169-2607(89)90159-4

[27] Hayes MJ, Smith PR (2001) A new method for pulse oximetry possessing inherent insensitivity to artifact. IEEE Trans Biomed Eng 48: 452–461 10.1109/10.915711 11322533

[28] Clark JW, Neuman MR, Olson WH, Peura RA, Primiano FP, et al. (2009) Medical Instrumentation Application and Design; John GW, editor. NJ: Wiley. 713 p.

[29] Apple. (2011) AV Foundation Programming Guide (iOS Developer Library). Available: http://developer.apple.com/library/ios/DOCUMENTATION/AudioVideo/Conceptual/AVFoundationPG/Articles/00_Introduction.html. Accessed 17 Feb 2014.

[30] Futran ND, Stack BC Jr, Hollenbeak C, Scharf JE (2000) Green light photoplethysmography monitoring of free flaps. Arch Otolaryngol Head Neck Surg 126: 659–662 10.1001/archotol.126.5.659 10807336

[31] Ludbrook J (1997) Comparing methods of measurements. Clin Exp Pharmacol Physiol 24: 193–203 10.1111/j.1440-1681.1997.tb01807.x 9075596

[32] Bland JM, Altman DG (1986) Statistical methods for assessing agreement between two methods of clinical measurement. Lancet 1: 307–310 10.1016/S0140-6736(86)90837-8 2868172

[33] Lindberg LG, Oberg PA (1991) Photoplethysmography. Part 2. Influence of light source wavelength. Med Biol Eng Comput 29: 48–54 10.1007/BF02446295 2016920

[34] Ugnell H, Oberg PA (1995) The time-variable photoplethysmographic signal; dependence of the heart synchronous signal on wavelength and sample volume. Med Eng Phys 17: 571–578 10.1016/1350-4533(95)00008-B 8564151

[35] Obeid AN, Boggett DM, Barnett NJ, Dougherty G, Rolfe P (1988) Depth discrimination in laser Doppler skin blood flow measurement using different lasers. Med Biol Eng Comput 26: 415–419. 3255852 10.1007/BF02442302

[36] Tanaka G, Sawada Y (2003) Examination of normalized pulse volume-blood volume relationship: toward a more valid estimation of the finger sympathetic tone. Int J Psychophysiol 48: 293–306 10.1016/S0167-8760(03)00056-4 12798989

[37] Magnenat-Thalmann N, Kalra P, Leveque JL, Bazin R, Batisse D, et al (2002) A computational skin model: fold and wrinkle formation. IEEE Trans Inf Technol Biomed 6: 317–323 10.1109/TITB.2002.806097 15224846

[38] Krishnan R, Natarajan BB, Warren S (2010) Two-stage approach for detection and reduction of motion artifacts in photoplethysmographic data. IEEE Trans Biomed Eng 57: 1867–1876 10.1109/TBME.2009.2039568 20172800

[39] Elgendi M, Norton I, Brearley M, Abbott D, Schuurmans D (2013) Systolic peak detection in acceleration photoplethysmograms measured from emergency responders in tropical conditions. PLoS One 8: e76585 10.1371/journal.pone.0076585 24167546 PMC3805543

